# Reproductive performance of gilthead seabream (*Sparus aurata*) broodstock showing different expression of fatty acyl desaturase 2 and fed two dietary fatty acid profiles

**DOI:** 10.1038/s41598-020-72166-5

**Published:** 2020-09-23

**Authors:** Shajahan Ferosekhan, Hanlin Xu, Serhat Turkmen, Ana Gómez, Juan Manuel Afonso, Ramon Fontanillas, Grethe Rosenlund, Sadasivam Kaushik, Marisol Izquierdo

**Affiliations:** 1 Aquaculture Research Group (GIA), IU-ECOAQUA, Universidad de Las Palmas de Gran Canaria, Las Palmas, Canary Islands Spain; 2 ICAR-Central Institute of Freshwater Aquaculture, Bhubaneswar, Odisha India; 3 Department of Biology, University of Alabama at Birmingham, Birmingham, USA; 4 Institute of Aquaculture Torre de La Sal (IATS), CSIC, Ribera de Cabanes, Castellón, Spain; 5 Skretting Aquaculture Research Centre, Stavanger, Norway

**Keywords:** Zoology, Lipids

## Abstract

Previous studies have shown that it is possible to nutritionally program gilthead seabream offspring through fish oil (FO) replacement by vegetable oils (VO) in the broodstock diet, to improve their ability to grow fast when fed low fish meal (FM) and FO diets during grow-out phase. However, in those studies broodstock performance was reduced by the VO contained diet. Therefore, the present study aimed to determine if it is possible to replace FO by a mixture of FO and rapeseed oil (RO) with a specific fatty acid profile in broodstock diets, without altering gilthead seabream broodstock reproductive performance. Besides, the study also aimed to evaluate the reproductive performance of broodstock with different expression of fatty acid desaturase 2 gene (*fads2)* a key enzyme in synthesis of long chain polyunsaturated fatty acids. For that purpose, broodfish having either a high (HD) or low (LD) expression of *fads2* were fed for three months during the spawning season with two diets containing different fatty acid profiles and their effects on reproductive hormones, fecundity, sperm and egg quality, egg biochemical composition and *fads2* expression were studied. The results showed that blood *fads2* expression in females, which tended to be higher than in males, was positively related to plasma 17β-estradiol levels. Moreover, broodstock with high blood *fads2* expression showed a better reproductive performance, in terms of fecundity and sperm and egg quality, which was correlated with female *fads2* expression. Our data also showed that it is feasible to reduce ARA, EPA and DHA down to 0.43, 6.6 and 8.4% total fatty acids, respectively, in broodstock diets designed to induce nutritional programming effects in the offspring without adverse effects on spawning quality. Further studies are being conducted to test the offspring with low FM and FO diets along life span.

## Introduction

Sustainable development of aquaculture depends much on the efficient use of two limited resources derived from capture fisheries: fishmeal (FM) and fish oil (FO)^1–3^. Great advances have been made to completely replace FM by alternative plant protein sources in diets for both freshwater and marine fish^1,4–8^. However, total replacement of FO in the diets of marine fish is difficult due to the scarcity of other sources of long chain polyunsaturated fatty acids (LC-PUFA) of the n-3 family, which are essential for purely marine teleosts^9,10^. Vegetable oils (VO) have been frequently used to partially replace FO, alone^11,12^ or in combination with FM replacement^13–16^. Replacement of FO by VO is constrained by the lack of n-3 LC-PUFA in VO, despite their high concentrations in 18 carbon (18C) fatty acid precursors, because marine teleost has a limited capacity of bioconversion of 18C fatty acids into LC-PUFA. The first step of n-3 LC-PUFA synthesis in fish is catalysed by delta 6 fatty acid desaturase (Δ6 Fads), which inserts an extra double bond in the precursors, linoleic acid (LA, 18:2n-6) or alpha-linolenic acid (ALA, 18:3n-3). Therefore, this enzyme produces 18:3n-6 and 18:4n-3 and, subsequently, longer carbon chain fatty acids, such as eicosapentaenoic acid (EPA, 20:5n-3) and docosahexaenoic acid (DHA, 22:6n-3) after several elongation, desaturation and β-oxidation steps^17,18^. However, marine fish have a low expression of *fads2*, the gene that codes for Δ6 Fads and, therefore, LC-PUFA must be included in their diet.

In the past years, novel lipid sources high in n-3 LC-PUFA have been studied, such as krill oil^19,20^, microalgae^21–25^ or the transgenic plant *Camelina sativa*^26,27^. However, these n-3 LC-PUFA sources are either still expensive or not produced at sufficient amounts to allow complete replacement of FO in diets for marine fish. One way to optimize the use of these novel lipid sources to completely replace FO would be to combine them with VO high in the 18C precursors and produce marine fish with a higher capacity of LC-PUFA biosynthesis. Two different approaches can be followed to modify LC-PUFA biosynthesis capacity in fish. On the one hand, nutrition during very early life history can markedly affect the ability of organisms to effectively utilize specific nutrients later in life, an effect that is known as nutritional programming or conditioning^28^. On the other hand, nutritional manipulation of broodstock diet or selection of broodstock for specific markers of lipid biosynthesis can alter the LC-PUFA biosynthetic capacity^29^. As the gilthead seabream (*Sparus aurata*) (GSB) is a multi-batch spawner, eggs largely depend on the continuous intake of nutrients to complete vitellogenesis during the whole spawning season. Therefore, adequate amounts of essential nutrients, most importantly LC-PUFA must be provided in broodstock diets for the proper gonadal and embryo development^30^.

Our previous studies have shown that conditioning GSB through specific broodstock diets produces juveniles and adults with a better ability to utilize low FM and FO diets and faster growth^29,31–33^. In this study, GSB broodstock were fed with four different levels of fatty acid precursors ratio (LA + ALA:n-3 LC-PUFA ratio levels, 7.96:20.52; 32.59:13.13; 41.99:10.60 and 39.97:9.48% total fatty acid)^32,33^. Dietary FO replacement by linseed oil (LO) up to 60% in GSB broodstock diet did not affect the reproductive performance, but further replacement of FO by linseed oil up to 80–100% in broodstock diets for gilthead seabream significantly reduced fecundity, larval quality, and growth of 45 days old fingerlings and 4-month-old juveniles^32,33^. However, offspring from broodstock fed the 100% FO diet showed the best growth and feed utilization even when fed low FM and low FO diets^32,33^. Therefore, it is necessary to find out the optimum levels that allow a nutritional programming effect to improve offspring growth without altering broodstock reproductive performance.

Preliminary studies suggest that broodstock with higher *fads2* expression show a higher fecundity than lower *fads2* expressed broodstock (Turkmen et al., in prep), but their specific effect in reproductive success has not been studied in detail. For instance, *FADS2* expression in mammals shows a positive relation with reproductive hormones, such as progesterone or estradiol^34^. In mice, *fads2* knocked-out lead to an impaired reproductive performance, eventually leading to failure in offspring production^35^. However, there are no specific studies in fish relating the *fads2* expression in broodstock and reproductive hormone levels, reproductive performance or the *fads2* expression in the eggs produced.

**Figure 1:**
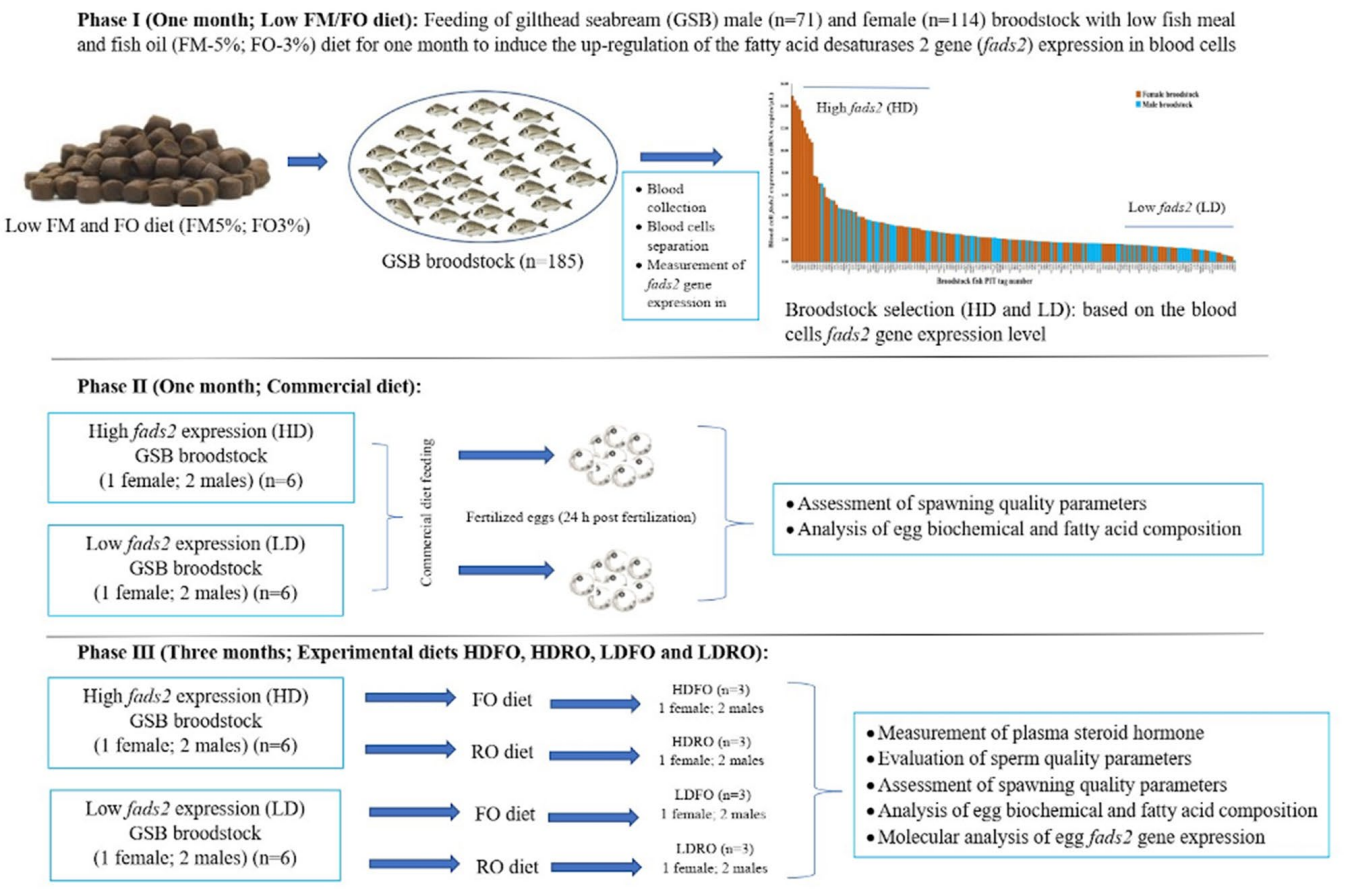
Schematic diagram of the study on broodstock selection (*fads2*) and dietary fatty acids profile on reproductive performance in gilthead seabream.

The present study aimed to determine if it is possible to replace FO by a mixture of VO and FO that provides LA + ALA: n-3 LC-PUFA ratio levels of 16.1:16.3 in broodstock diets, without altering gilthead seabream broodstock reproductive performance. Secondly, it also aimed to determine the reproductive performance of broodstock with different *fads2* expression levels and the potential interaction with the broodstock diet. For that purpose, broodfish having either a high (HD) or low (LD) expression of *fads2* were fed for three months during the spawning season with two diets containing different fatty acid profiles. Different parameters of reproductive performance such as plasma steroid hormone levels, fecundity, sperm, and egg quality were recorded, together with the egg biochemical and fatty acid composition and *fads2* expression. The schematic diagram of experimental design is presented in Fig. 1.

## Results

### Characterization of broodstock with high or low *fads2* expression (Phase-I)

**Figure 2:**
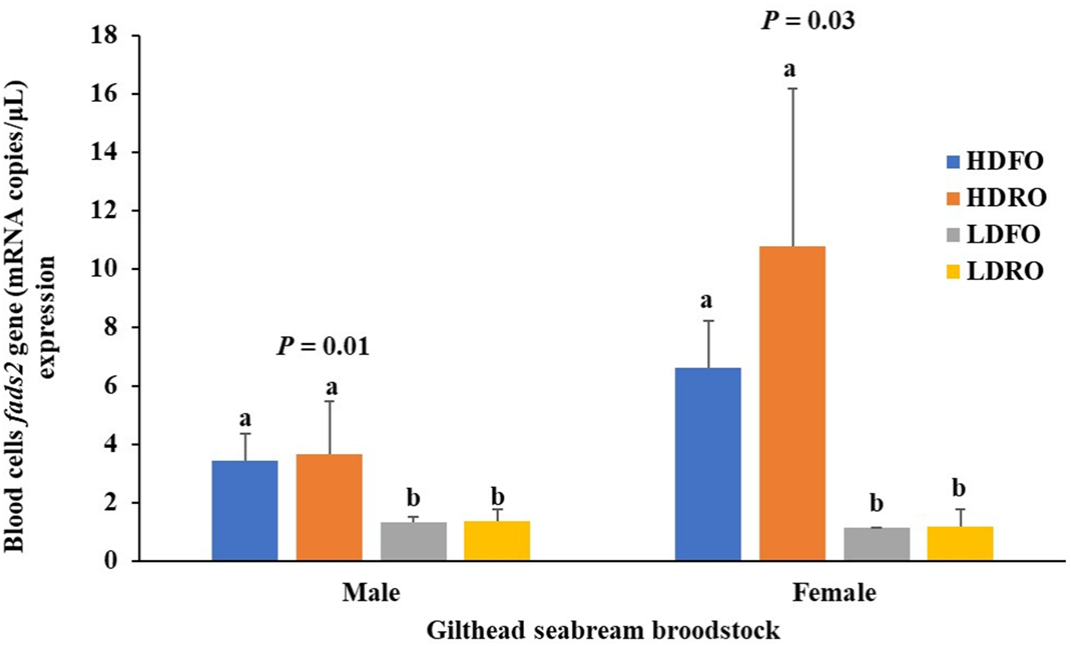
Blood cells *fads2* gene (mRNA copies/μL) expression of gilthead seabream male (n = 12) and female (n = 6) broodstock selected at the end of Phase I and assigned to different groups (HDFO, HDRO, LDFO and LDRO) for subsequent studies. Different superscripts in male or female mean bar indicate significant differences (*P* < 0.05, one-way ANOVA, Tukey Post-Hoc test).

Broodstock body weight was not related to the *fads2* expression levels and HD and LD broodstock had similar body weight (*P* > 0.05) before the experimental period (Table 1). The *fads2* expression values in males and females broodstock were in the range of 0.15–7.06 and 0.49–14.92 copies/µl, respectively (Supplementary file, Fig. S1). The mean blood cells *fads2* expression value for males (2.26 ± 3.17copies/µl, n = 71) was found to be 32% lower than that of females (3.31 ± 3.16 copies/µL, n = 114) for all the selected broodstock (data not shown). Besides, around 10% of the total female broodstock exhibited higher *fads2* (> 7.06 copies/µL) expression than any male. In total, 6 females and 12 males with high (HD) or low (LD) *fads2* expression were selected from the highest and lowest *fads2* expression broodstock, their mean values are shown in Fig. 2. The body weight of the female and male fish which were selected for the next phase (Phase II) showed no significant differences (Table 1).

**Table 1:** Body weight (kg) of gilthead seabream male (n = 6) and female (n = 3) broodstock selected based on blood cells *fads2* expression at the end of Phase I.

Body weight (kg)	HDFO	HDRO	LDFO	LDRO	*P* value
Male	0.92 ± 0.15	0.85 ± 0.18	0.94 ± 0.16	0.96 ± 0.16	0.73
Female	2.34 ± 0.11	2.29 ± 0.31	2.09 ± 0.23	2.22 ± 0.35	0.83

Different superscripts in a line indicate significant differences among broodfish groups for a given parameter (*P* < 0.05, one-way ANOVA, Tukey Post-Hoc test).

### Comparison of broodstock quality before feeding the conditioning diets (Phase-II)

After one month of feeding the commercial broodstock diet (Phase II) at the beginning of the spawning season, no significant differences (*P* > 0.05) were found in any of the spawning quality parameters tested among broodfish from the same category (HD and LD) (Supplementary file, Table S1). Besides, eggs proximate (Table S2) and fatty acid composition (Tables S3) also did not show significant (*P* > 0.05) differences.

### Broodstock nutritional conditioning (Phase-III)

#### Plasma steroid hormones

Analysis of plasma steroid hormones after feeding the experimental diets denoted no significant differences in plasma testosterone, 11-ketotestosterone or 17β-estradiol levels among the different broodstock groups (Table 2). The highest 11-ketotestosterone levels were found in broodstock with the smallest weight and a significant negative linear regression relation was found between broodstock body weight and 11-ketotestosterone levels (R^2^ = 0.517; *P* < 0.001) (Fig. S2a). The opposite relation was found with the 17β-estradiol levels that showed a significant positive exponential relation with the broodstock body weight (R^2^ = 0.464; *P* < 0.001) (Fig. S2b). No relation was found between plasma steroid hormones and broodstock blood cells *fads2* expression when all broodstock data were compared. However, in females with low *fads2* expression, there was a positive linear regression relation but no significant difference between plasma 17β-estradiol levels and blood cells *fads2* expression (R^2^ = 0.502; *P* = 0.115) was observed.

**Table 2:** Plasma steroid hormone levels of male (n = 12) and female (n = 6) gilthead seabream broodstock with high (HD) or low (LD) *fads2* expression fed with either FO or RO experimental diets during Phase-III.

Plasma steroid hormones (ng/ml)	Sex	HDFO	HDRO	LDFO	LDRO	*P* value
Testosterone	Male	0.341 ± 0.064	0.482 ± 0.221	0.562 ± 0.216	0.418 ± 0.197	0.55
	Female	1.074 ± 1.138	0.332 ± 0.414	0.252 ± 0.093	0.285 ± 0.011	0.54
11 Keto-testosterone	Male	0.059 ± 0.049	0.073 ± 0.026	0.071 ± 0.029	0.089 ± 0.022	0.55
	Female	0.039 ± 0.049	0.011 ± 0.006	0.008 ± 0.001	0.006 ± 0.000	0.57
17β-estradiol	Male	0.434 ± 0.467	0.196 ± 0.081	0.332 ± 0.187	0.185 ± 0.055	0.29
	Female	3.044 ± 1.107	1.019 ± 0.985	1.444 ± 0.943	1.590 ± 1.080	0.35

Different superscripts in a line would indicate significant differences among broodfish groups for a given parameter (*P* < 0.05, one-way ANOVA, Tukey Post-Hoc).

#### Sperm quality

**Figure 3:**
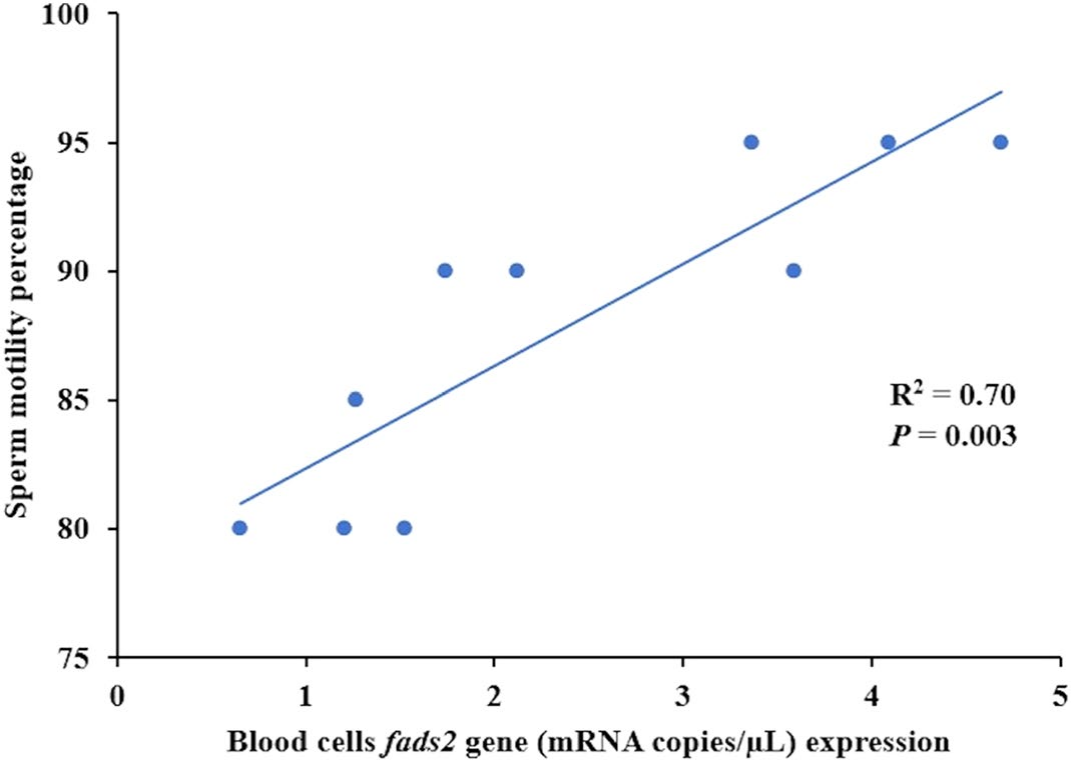
The relationship between blood cells *fads2* expression (end of Phase I) and sperm motility percentage in male (n = 10) gilthead seabream of the high or low *fads* groups and fed either the fish oil (FO) or the rapeseed oil (RO) diets measured at the end of Phase III.

After one month of feeding the experimental conditioning diets, sperm concentration or motility duration were not influenced by the *fads2* expression in the broodstock neither by the dietary fatty acids profile or their combination as denoted by the two-way ANOVA analysis (*P* > 0.05) (Table 3). However, the percentage of sperm showing motility after activation with seawater was around 10% higher in broodstock with higher *fads2* expression, indicating the effect of *fads2* expression in broodstock on sperm motility (Table 3). Indeed, a highly significant positive linear regression relation was found between *fads2* expression levels in broodstock males and sperm motility (R^2^ = 0.70; *P* = 0.003) (Fig. 3). Besides, a positive linear regression relation but no significant difference was observed between the male testosterone levels and the sperm concentration (R^2^ = 0.823; *P* = 0.093). No other relation was found between sperm quality and male hormones plasma levels.

**Table 3:** Sperm quality (n = 6) from the different gilthead seabream broodstock groups fed either the FO or the RO experimental diets (Phase-III).

Sperm quality parameters	Broodfish groups				Two-way ANOVA *P* values		
	HDFO	HDRO	LDFO	LDRO	Broodstock *fads2* expression	Diet	Broodstock *fads2* expression x Diet
Sperm concentration (10^9^ sperm/ml)	8.63 ± 1.22	9.96 ± 0.60	11.12 ± 0.23	9.28 ± 2.34	0.38	0.80	0.15
Spermatocrit percentage	77.50 ± 3.54	70.00 ± 13.23	55.00 ± 0.00	71.67 ± 7.64	0.12	0.46	0.08
Sperm motility percentage	92.50 ± 3.54	93.33 ± 2.89	82.50 ± 3.54	83.33 ± 5.77	0.01	0.77	1.00
Sperm motility duration (Second)	810 ± 127	780 ± 312	630 ± 127	760 ± 277	0.56	0.77	0.64

No significant differences for sperm quality parameters among broodfish groups were observed (*P* < 0.05, one-way ANOVA, Tukey Post-Hoc).

#### Egg and larval quality

**Figure 4:**
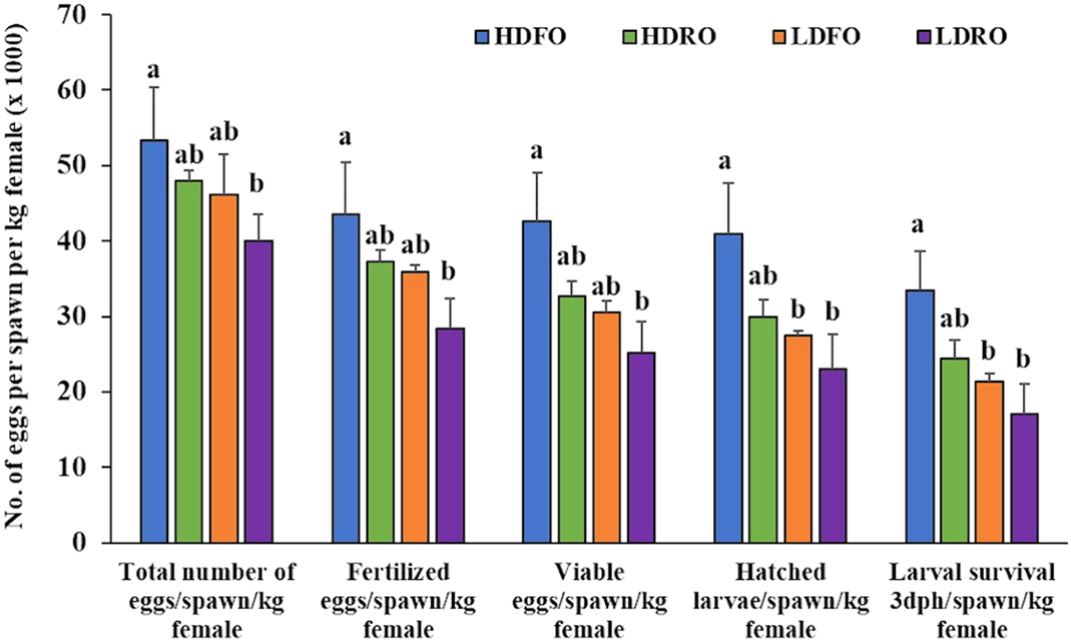
Reproductive performance of gilthead seabream broodstock (n = 76 spawns over three months) with high (HD) or low (LD) *fads2* expression fed with either FO or RO diet over three months (Phase-III). Different superscript letters for each spawning quality parameters differ significantly (*P* < 0.05, One-way ANOVA, Tukey Post-Hoc).

**Figure 5:**
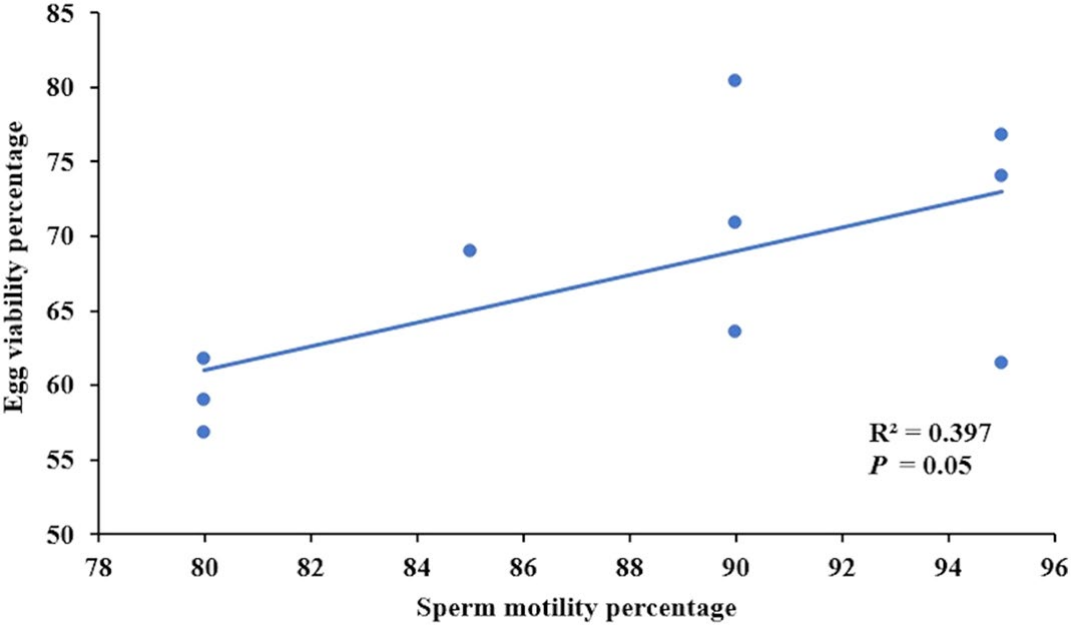
The relationship between sperm motility and egg viability percentage (n = 10) of the different groups (HDFO, HDRO, LDFO and LDRO) of gilthead seabream broodstock, assessed at the end of Phase-III.

**Figure 6:**
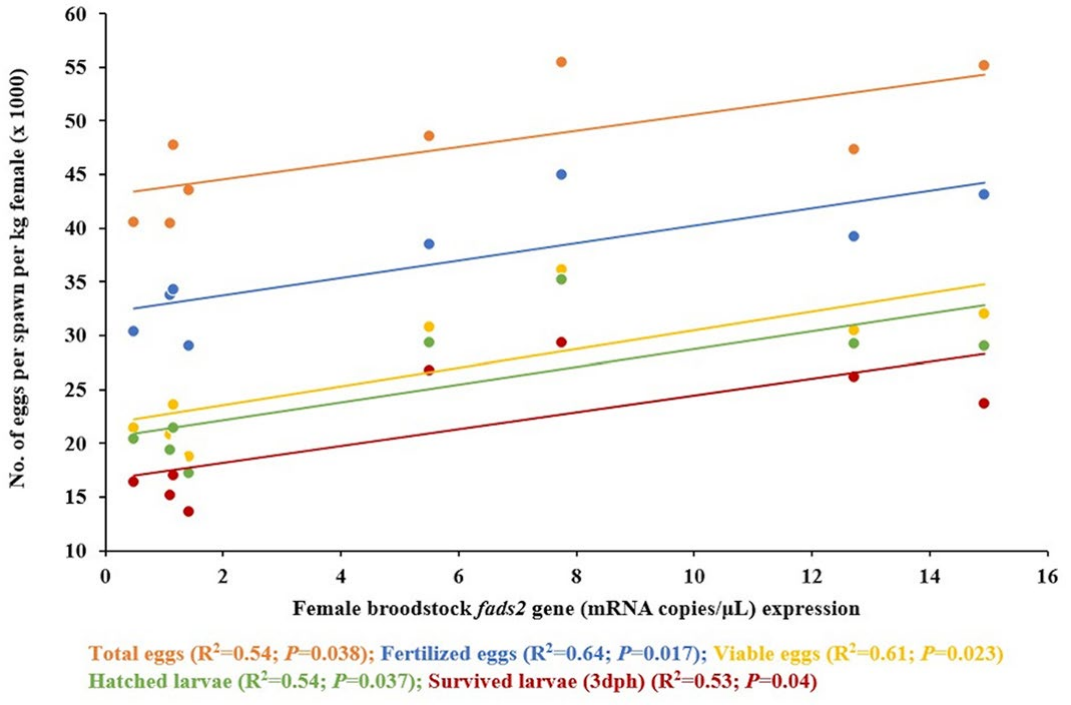
The linear regression relationship between female blood cells *fads2* expression (measured at the end of the preparatory Phase I) and spawning quality (n = 8) of the different groups (HDFO, HDRO, LDFO and LDRO) of gilthead seabream broodstock over the whole Phase III.

Before feeding the experimental conditioning diets neither broodstock quality nor egg biochemical composition differed among experimental broodstock (Table S1, S2 and S3). However, after feeding the experimental diets, fish fecundity, measured as mean number of eggs/spawn/kg female, was the highest in HDFO broodstock and the lowest in LDRO broodstock (Fig. 4; Table 4). Thus, the two-way ANOVA analysis showed a significant (*P* < 0.05) improvement in fecundity in broodstock with higher *fads2* expression, whereas feeding RO did not significantly (*P* > 0.05) affected broodstock fecundity. Other spawning quality parameters such as fertilization, hatching or larval survival rates were not affected by neither broodstock *fads2* expression nor by the broodstock conditioning diet or their combination (Table 4). Only egg viability rate was slightly improved in broodstock with higher *fads2* expression (*P* = 0.07) (Table 4). Besides, a significant linear relation (R^2^ = 0.397; *P* = 0.05) was found between sperm motility and egg viability percentage (Fig. 5). A significant linear regression relationship was observed between female broodstock blood cells *fads2* expression and all the spawning quality parameters (Fig. 6). No interaction between diet or broodstock *fads2* expression was detected by the two-way ANOVA analysis (Table 4). As a consequence, the number of fertilized eggs/spawn/kg female (*P* < 0.05) and particularly, the numbers of viable eggs/spawn/kg female, hatched larvae/spawn/kg female or larval survival 3 dph/spawn/kg female were the highest in HDFO broodstock and the lowest in LDRO (Fig. 4).

**Table 4:** Quality of egg and larvae (n = 76 spawns per three months) obtained from the different gilthead seabream broodstock groups fed either the FO or the RO experimental diets (Phase-III).

Egg and larval quality parameters	Broodfish groups				Two-way ANOVA *P* values		
	HDFO	HDRO	LDFO	LDRO	Broodstock *fads2* expression	Diet	Broodstock *fads2* expression x Diet
Nº of eggs/spawn/kg female	53,398 ± 6,943^a^	48,040 ± 1323^ab^	46,086 ± 5458^ab^	40,044 ± 3464^b^	0.03	0.08	0.90
Fertilization %	80.34 ± 2.57	76.19 ± 6.21	77.50 ± 12.51	70.51 ± 6.73	0.47	0.29	0.72
Egg viability %	78.61 ± 2.55	66.35 ± 6.73	65.39 ± 5.06	62.22 ± 7.60	0.07	0.09	0.26
Hatching %	96.04 ± 1.47	90.23 ± 4.11	89.50 ± 2.41	90.66 ± 3.76	0.15	0.27	0.13
Larval survival (3dph) %	81.72 ± 0.59	76.27 ± 5.43	75.69 ± 2.23	71.96 ± 6.66	0.14	0.21	0.71

Different superscripts in a line indicate significant differences among broodfish groups for a given parameter (*P* < 0.05, one-way ANOVA, Tukey Post-Hoc).

#### Egg biochemical composition

**Figure 7:**
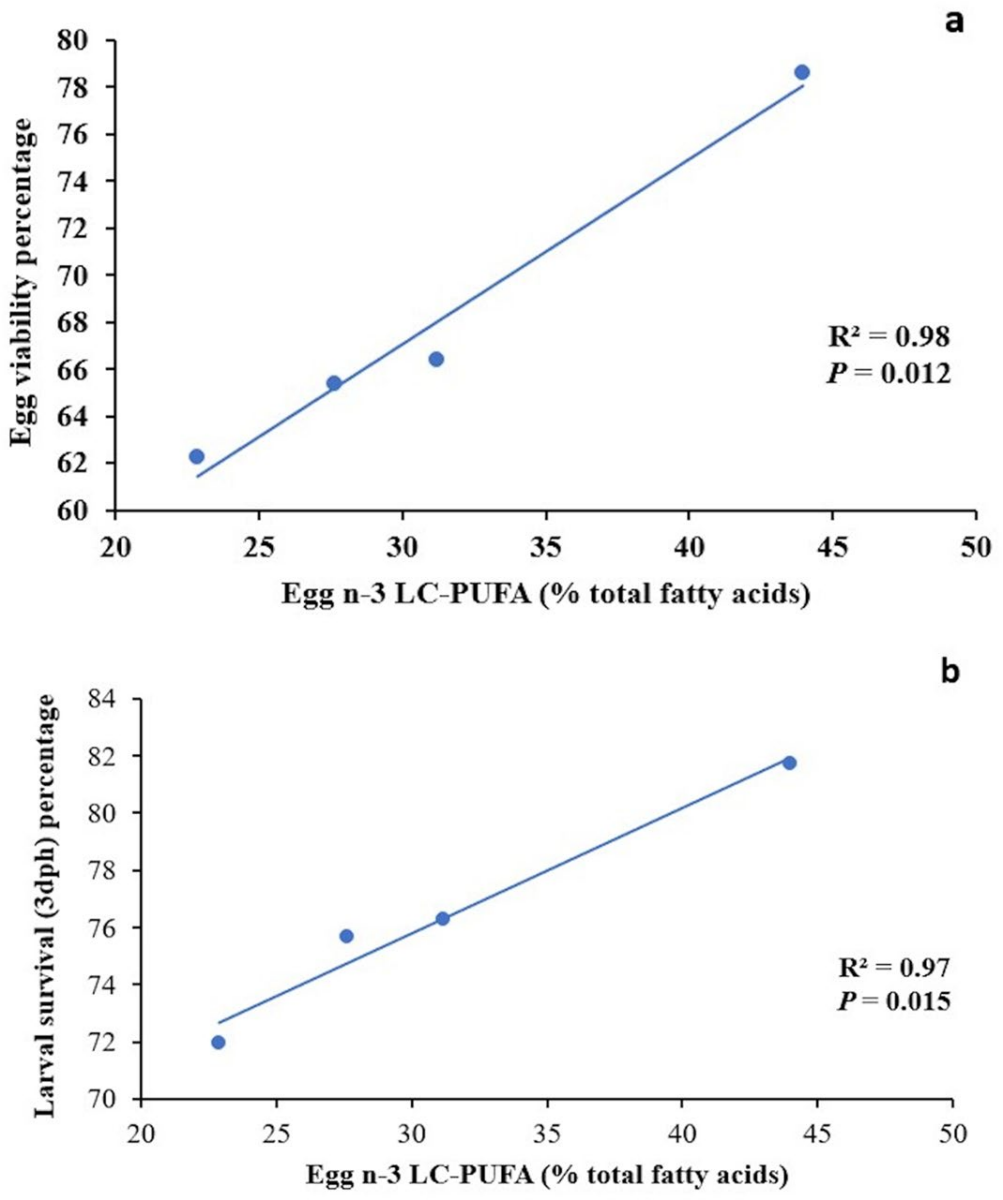
Effect of eggs n-3 LC-PUFA contents (one month after experimental diet feeding) and egg viability (**a**) or larval survival at 3 dph (**b**) (n = 4) measured over the whole Phase III.

Proximate composition of eggs was not significantly (*P* > 0.05) affected either by the *fads2* expression in broodstock or the experimental conditioning diet (Table 5). However, the two-way ANOVA analysis showed that eggs from broodstock with a higher *fads2* expression (HDFO and HDRO) showed a significant (*P* < 0.05) increase in 20:3n-9, 22:5n-3, DHA/ARA, n-3/n-6, and, particularly, 22:6n-3 (40% average increase), and a mild (*P* < 0.10) increase in 16:3n-3, 16:4n-3, 18:4n-3 and DHA/EPA (Table 6). Besides, the relative contents in 14:0, 14:1n-7, 14:1n-5, 15:0, 15:1n-5, 16:0 ISO, 16:0, 16:1n-7, 16:1n-5, 16:2n-4, 17:0, 18:1n-9, 18:2n-4 and 22:4n-6 were reduced in eggs from broodstock with a higher *fads2* expression (Table 6). As expected from their dietary levels, the two-way ANOVA analysis showed that feeding broodstock with RO diet (HDRO and LDRO) significantly (*P* < 0.05) increased 18:1n-9, 18:2n-6, and 18:3n-3 (60% average increase) as well as total monounsaturated and n-6 fatty acids (Table 6). Besides, feeding RO diet reduced (*P* < 0.05) the levels of 16:1n-7, 17:0, 20:4n-6, 20:5n-3, 22:6n-3 (30% average reduction) as well as those of total saturated, n-3 and n-3/n-6 fatty acids (Table 6). Both egg viability (Fig. 7a) and larval survival (Fig. 7b) at 3 dah showed highly significant positive linear regression relationship to the total n-3 LC-PUFA content in the egg (y = 0.787x + 43.423, R^2^ = 0.98, *P* = 0.012; and y = 0.4384x + 62.639, R^2^ = 0.97, *P* = 0.015, respectively).

**Table 5:** Biochemical composition (n = 3) of gilthead seabream eggs obtained from the different broodstock groups fed either the FO or the RO experimental diets (Phase-III).

Egg biochemical composition	Broodfish groups				Two-way ANOVA *P* values		
	**HDFO**	**HDRO**	**LDFO**	**LDRO**	**Broodstock** ***fads2*** *expression*	**Diet**	**Broodstock** ***fads2*** **expression x Diet**
Crude protein (% DM)	77.23 ± 0.51	72.74 ± 1.77	74.18 ± 4.49	73.73 ± 2.17	0.46	0.17	0.22
Crude lipid (% DM)	21.48 ± 2.11	21.71 ± 1.21	22.45 ± 2.60	23.88 ± 3.26	0.34	0.67	0.89
Moisture (%)	89.09 ± 1.29	89.00 ± 3.26	91.77 ± 0.10	90.78 ± 0.64	0.17	0.75	0.67

No significant differences for eggs biochemical composition among broodfish groups were observed (*P* < 0.05, one-way ANOVA, Tukey Post-Hoc).

**Table 6:** Fatty acid composition (expressed as % total fatty acids) of gilthead seabream eggs (n = 3) from the different broodstock groups fed either the FO or the RO experimental diets (Phase-III).

Fatty acids (%TFA)	Broodfish groups				Two-way ANOVA *P* values		
	**HDFO**	**HDRO**	**LDFO**	**LDRO**	**Broodstock** ***fads2*** *expression*	**Diet**	**Broodstock** ***fads2*** **expression x Diet**
14:0	1.48 ± 0.11	1.18 ± 0.12	3.85 ± 0.17	3.43 ± 1.71	0.01	0.60	0.93
14:1n-7	0.04 ± 0.01^ab^	0.02 ± 0.02^b^	0.11 ± 0.02^ab^	0.16 ± 0.06^a^	0.01	0.47	0.20
14:1n-5	0.07 ± 0.01	0.05 ± 0.01	0.17 ± 0.01	0.23 ± 0.11	0.02	0.64	0.43
15:0	0.21 ± 0.01^b^	0.17 ± 0.01^b^	0.43 ± 0.01^a^	0.38 ± 0.08^a^	0.00	0.18	0.82
15:1n-5	0.04 ± 0.01^b^	0.03 ± 0.01^b^	0.13 ± 0.01^ab^	0.21 ± 0.06^a^	0.00	0.19	0.08
16:0 ISO	0.10 ± 0.01^b^	0.04 ± 0.00^b^	0.09 ± 0.01^b^	0.20 ± 0.05^a^	0.01	0.17	0.00
16:0	12.11 ± 0.49^b^	12.10 ± 0.92^b^	19.74 ± 0.78^a^	15.14 ± 2.29^ab^	0.00	0.05	0.05
16:1n-7	3.71 ± 0.57^bc^	2.73 ± 0.07^c^	5.69 ± 0.08^a^	3.83 ± 0.41b	0.00	0.00	0.09
16:1n-5	0.13 ± 0.04^b^	0.07 ± 0.02^b^	0.16 ± 0.04^b^	0.31 ± 0.07^a^	0.01	0.19	0.01
16:2n-4	0.27 ± 0.02	0.16 ± 0.05	0.39 ± 0.06	0.34 ± 0.12	0.03	0.18	0.65
17:0	0.24 ± 0.01^ab^	0.13 ± 0.02^b^	0.35 ± 0.01^a^	0.28 ± 0.08^a^	0.01	0.02	0.55
16:3n-4	0.26 ± 0.01	0.18 ± 0.01	0.31 ± 0.01	0.56 ± 0.28	0.08	0.43	0.17
16:3n-3	0.15 ± 0.01	0.11 ± 0.01	0.18 ± 0.01	0.42 ± 0.21	0.08	0.25	0.13
16:3n-1	0.13 ± 0.01	0.10 ± 0.01	0.17 ± 0.04	0.39 ± 0.23	0.11	0.30	0.20
16:4n-3	0.18 ± 0.01	0.14 ± 0.02	0.27 ± 0.04	0.46 ± 0.23	0.06	0.39	0.23
18:0	3.94 ± 0.42	3.60 ± 0.07	4.78 ± 0.66	3.63 ± 0.72	0.25	0.08	0.28
18:1n-9	16.16 ± 2.03	26.15 ± 1.74	16.79 ± 1.62	19.34 ± 5.01	0.19	0.02	0.13
18:1n-7	2.84 ± 0.24	2.95 ± 0.24	3.18 ± 0.16	5.03 ± 1.35	0.06	0.11	0.14
18:1n-5	0.19 ± 0.01	0.14 ± 0.02	0.23 ± 0.01	0.82 ± 0.74	0.17	0.28	0.22
18:2n-9	0.11 ± 0.01^b^	0.09 ± 0.02^b^	0.22 ± 0.07^ab^	0.37 ± 0.13^a^	0.01	0.24	0.15
18:2n-6	5.49 ± 1.03^b^	10.59 ± 0.41^a^	6.50 ± 1.12^b^	8.11 ± 1.53^ab^	0.34	0.00	0.05
18:2n-4	0.21 ± 0.00^ab^	0.11 ± 0.01^b^	0.25 ± 0.03^ab^	0.38 ± 0.13^a^	0.02	0.74	0.05
18:3n-6	0.23 ± 0.01	0.19 ± 0.04	0.37 ± 0.02	0.79 ± 0.42	0.06	0.27	0.19
18:3n-4	0.23 ± 0.05^ab^	0.12 ± 0.02^b^	0.21 ± 0.03^ab^	0.42 ± 0.13^a^	0.03	0.32	0.02
18:3n-3	1.21 ± 0.35^b^	2.73 ± 0.34^a^	1.34 ± 0.16^b^	2.23 ± 0.38^ab^	0.43	0.00	0.20
18:4n-3	0.82 ± 0.11	0.48 ± 0.08	0.94 ± 0.13	1.10 ± 0.43	0.07	0.62	0.19
18:4n-1	0.19 ± 0.01	0.08 ± 0.01	0.19 ± 0.03	0.38 ± 0.32	0.24	0.73	0.26
20:0	0.20 ± 0.02	0.16 ± 0.03	0.19 ± 0.03	0.51 ± 0.31	0.20	0.27	0.18
20:1n-9	0.27 ± 0.02	0.22 ± 0.03	0.24 ± 0.01	0.35 ± 0.21	0.55	0.67	0.35
20:1n-7	1.15 ± 0.06	1.37 ± 0.25	1.00 ± 0.15	0.88 ± 0.36	0.11	0.78	0.37
20:1n-5	0.25 ± 0.04	0.17 ± 0.03	0.29 ± 0.07	0.40 ± 0.33	0.31	0.87	0.49
20:2n-9	0.10 ± 0.04	0.07 ± 0.01	0.16 ± 0.01	0.45 ± 0.41	0.20	0.43	0.33
20:2n-6	0.31 ± 0.01	0.42 ± 0.05	0.33 ± 0.02	0.59 ± 0.32	0.46	0.17	0.56
20:3n-9	0.07 ± 0.01	0.04 ± 0.01	0.09 ± 0.03	0.24 ± 0.13	0.05	0.21	0.10
20:3n-6	0.20 ± 0.03	0.16 ± 0.01	0.23 ± 0.03	0.80 ± 0.61	0.19	0.29	0.23
20:4n-6	1.35 ± 0.04	0.79 ± 0.05	1.25 ± 0.22	1.00 ± 0.38	0.74	0.04	0.34
20:3n-3	0.25 ± 0.04	0.29 ± 0.05	0.28 ± 0.01	0.58 ± 0.36	0.28	0.25	0.39
20:4n-3	0.87 ± 0.01	0.60 ± 0.08	0.69 ± 0.03	0.64 ± 0.27	0.55	0.19	0.34
20:5n-3	9.75 ± 0.58	5.75 ± 0.36	7.65 ± 0.45	6.00 ± 3.00	0.45	0.05	0.34
22:1n-11	0.51 ± 0.11	0.44 ± 0.06	0.46 ± 0.02	0.73 ± 0.66	0.65	0.70	0.52
22:1n-9	0.29 ± 0.09	0.21 ± 0.03	0.26 ± 0.09	0.71 ± 0.82	0.48	0.56	0.42
22:4n-6	0.18 ± 0.01^ab^	0.08 ± 0.02^b^	0.33 ± 0.04^ab^	0.85 ± 0.38^a^	0.02	0.19	0.07
22:5n-6	0.43 ± 0.04	0.24 ± 0.03	0.58 ± 0.01	0.90 ± 0.53	0.09	0.74	0.25
22:5n-3	3.75 ± 0.17^a^	2.79 ± 0.07^ab^	2.17 ± 0.10^b^	2.14 ± 0.56^b^	0.00	0.06	0.08
22:6n-3	29.35 ± 3.78^a^	21.75 ± 2.29^b^	16.83 ± 0.81^bc^	13.52 ± 0.83^c^	0.00	0.01	0.17
Ʃ Saturates	18.16 ± 0.16^b^	17.34 ± 1.11^b^	29.34 ± 1.56^a^	23.37 ± 3.18^ab^	0.00	0.04	0.10
Ʃ Monoenes	25.62 ± 2.83	34.55 ± 2.15	28.66 ± 1.68	32.73 ± 4.10	0.76	0.02	0.26
Ʃ n-3	46.32 ± 3.78^a^	34.63 ± 1.50^b^	30.34 ± 1.17^b^	27.08 ± 3.98^b^	0.00	0.01	0.07
Ʃ n-6	8.17 ± 0.92^b^	12.47 ± 0.44^a^	9.57 ± 0.85^ab^	13.05 ± 1.98^a^	0.28	0.00	0.64
Ʃ n-3 HUFA	43.97 ± 4.24^a^	31.18 ± 1.90^b^	27.62 ± 1.40^b^	22.87 ± 3.80^b^	0.00	0.00	0.09
DHA/EPA	3.01 ± 0.21	3.81 ± 0.65	2.20 ± 0.03	2.56 ± 0.92	0.05	0.22	0.62
DHA/ARA	21.71 ± 2.11^ab^	27.60 ± 1.20^a^	13.71 ± 1.76^b^	14.84 ± 5.28^b^	0.00	0.15	0.31
n-3/n-6	5.73 ± 1.10^a^	2.78 ± 0.19^b^	3.19 ± 0.40^b^	2.14 ± 0.63^b^	0.01	0.00	0.05

Superscripts in a line indicate significant differences in concentrations for a given fatty acid (*P* < 0.05, one-way ANOVA, Tukey Post-Hoc).

#### Molecular studies

**Figure 8:**
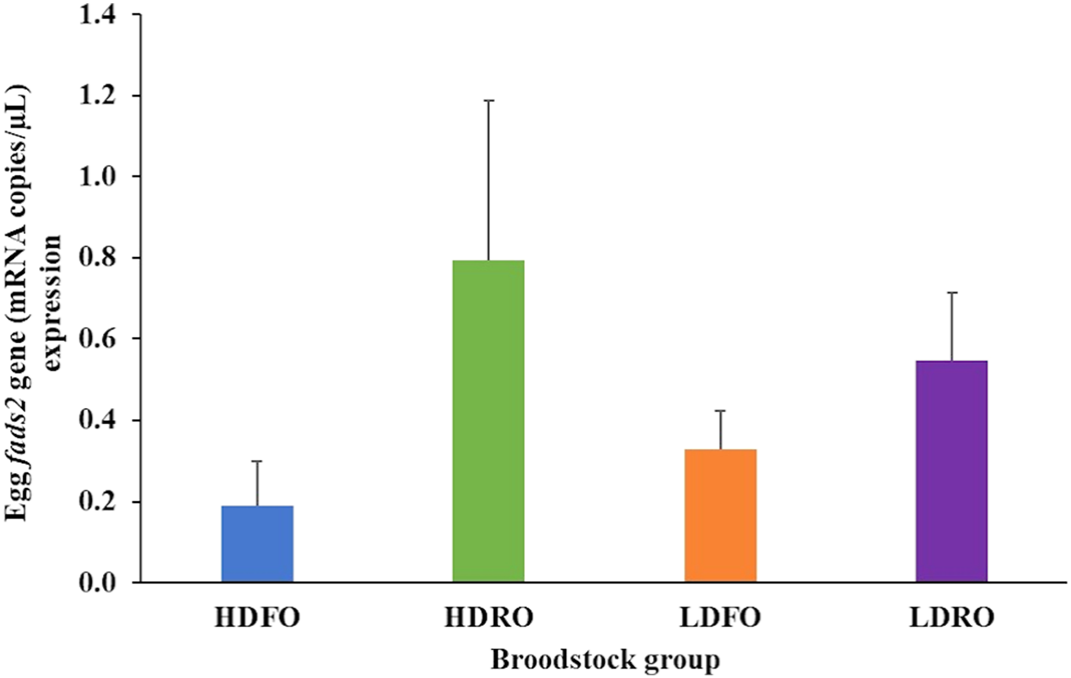
Expression of *fads2* in eggs (n = 3) of gilthead seabream broodstock of high (HD) or low (LD) *fads2* expression fed with either FO or RO experimental diets at the end of Phase-III.

The study of absolute *fads2* expression (mRNA copies/µl) in eggs from the different broodstock showed values that in average were 3 times higher for broodstock fed RO diet than for those fed FO (Fig. 8). However, due to the large standard deviations, there were not significant (*P* > 0.05) differences in *fads2* expression according to both the one-way or two-way ANOVA analysis.

## Discussion

Marine fish have a limited ability to synthesise LC-PUFAs, due to insufficient expression of key genes such as *fads2* and the inhibition of desaturase enzymes by dietary LC-PUFA^11,18,36–38^. Therefore, large FO replacement by VO in broodstock diets may lead to extremely low dietary LC-PUFA levels and markedly reduce broodstock performance^33,39^. Optimizing FO replacement by VO is desired to induce nutritional programming in the offspring for a better use of low FM and low FO diets^32,33^. Although, it has been found that there are large variations in the *fads2* expression showed by different gilthead seabream individuals, there is little information on its relation to fish reproductive performance^29^. The present study thus aimed to determine the reproductive success of gilthead seabream broodstock with different bloods *fads2* expression fed diets containing different types of oils.

Plasma 17β-estradiol and 11-ketotestosterone levels showed respectively positive and negative correlations to the body weight of broodstock GSB, as is expected from a protandric hermaphrodite species that turns from male to female as it ages^40^. The lack of relation between body weight and plasma testosterone levels as can be attributed to the fact that this hormone is an intermediate metabolite in most protandric fish^41^. Broodstock body weight had no effect on *fads2* expression, in males or females, nor with any parameter regarding spawning quality. The higher level of expression of *fads2* (30% more in females than in the males) and the significant positive correlation between plasma 17β-estradiol levels and the *fads2* expression in females from the low *fads2* expression groups are noteworthy. These results are in agreement with the increased conversion of LNA to n-3 LC-PUFA promoted by oestrogen in women, claimed to be important to fulfil the essential fatty acid requirements of the foetus and the neonate^42^. In fish, n-3 LC-PUFA are also highly demanded during embryogenesis and larval development to sustain growth, normal development and neural and sensorial organs formation, with EPA and particularly DHA being the major fatty acids found in marine fish eggs and larvae^9,43^. To our knowledge, this is the first study that finds a significant correlation between plasma oestrogen levels and *fads2* expression in blood cells of female fish, as observed in pregnant rats^34,44^. Indeed, treatments with 17β-estradiol have shown to increase *fads2* expression in female rats^45^. This higher *fads2* expression in females seems to be a response to the higher level of requirement of n-3 LC-PUFA fatty acid for the normal gonadal, oocyte and larval development through vitellogenetic processes in different teleosts^9,14,33,43,46,47^. Interestingly, in females showing a very high expression of *fads2* such a strong correlation was not found, despite a general trend to increased *fads2* expression in females with higher oestrogen levels. These results suggest the influence of a different genetic or epigenetic background between both types of females showing very high or low *fads2* expression.

Very early, it was reported that stenohaline marine teleosts have lower LC-PUFA bioconversion capacities compared to freshwater teleosts^48^. More recent work found that freshwater fish to possess greater number of copies of Fads2, compared to marine fish and that the Fads2 was a key metabolic gene for overcoming some of the nutritional constraints associated with freshwater environment^49^. We observed that there was a very large variation in the individual *fads2* expression values, for males the maximum value being 47 times higher than the minimum one and for females 30 times, in agreement with the large variation found in our previous studies^29^. From the 215 broodfish analysed for *fads2* expression, about 10% of the population had *fads2* expression values that were higher than 5 mRNA copies μL^−1^. In humans, *FADS2* expression and delta-6 desaturase activity is linked to different *FADS2* genotypes and, in turn to lower DNA methylation in specific CpG sites of the FADS2 promoter^50^. In agreement with human studies, in gilthead seabream, there is an increased methylation of specific CpG sites in the promoter region of this gene in offspring from broodstock with low *fads2* expression^29^. Moreover, LC-PUFA biosynthesis ability is affected by single nucleotide polymorphisms (SNPs) in the fatty acid desaturases^51,52^. Further studies are under way to elucidate the genetic and epigenetic mechanisms regulating *fads2* expression and LC-PUFA biosynthesis in gilthead seabream.

The improved reproductive performance in terms of sperm and egg quality in as observed here in broodstock with high blood *fads2* expression clearly reflects the importance of the end product(s) of *fads2* in fish reproduction. Sperm quality, in terms of motility and concentration, has been found to depend on its content in n-3 LC-PUFA, particularly DHA, in gilthead seabream after cryopreservation^53^, as well as in rainbow trout (*Oncorhynchus mykiss*)^54^, European seabass (*Dicentrarchus labrax*)^55^, Senegalese sole (*Solea senegalensis*)^56^ or European eel (*Anguilla Anguilla*)^57^ fed different amounts n-3 LC-PUFA. Therefore, a higher *fads2* expression in gilthead seabream broodstock would allow a higher n-3 LC-PUFA synthesis to promote a higher incorporation of these fatty acids into sperm that would have improved sperm motility, affecting in turn egg viability. Besides, testis fatty acids profile may also affect seminal plasma, which maintains sperm cells in a quiescent state required to achieve motility^54,58^. Finally, mice and human sperm also have high contents in DHA, which also acts as a precursor of very long chain LC-PUFAs with 26–32 carbons that form sphingolipids in spermatozoa and have been related to sperm quality^59^.

As regards seabream females where those with a higher *fads2* expression exhibited 20% higher fecundity in terms of eggs and larvae produced per kg female per spawn, this can also be linked to the production of n-3 LC-PUFA. Indeed, the DHA content in the eggs was effectively increased in females with a high *fads2* expression, denoting an efficient n-3 LC-PUFA biosynthesis in these females in comparison to those with low *fads2* expression, regardless of the diet fed. Interestingly, among the different LC-PUFA, only those from the n-3 series and with 22 carbons were significantly increased, despite the higher dietary content of 18:2n-6 (5.5–10.6% total fatty acid, TFA) in comparison to 18:3n-3 (1.2–2.7% TFA). Therefore, n-3/n-6 and DHA/ARA ratios were increased and 22:4n-6 reduced in females with a high *fads2* expression, suggesting the preference of the enzymatic complexes involved in fatty acid deposition in eggs for the n-3 LC-PUFA. The higher DHA/EPA and 22:5n-3 (n-3 DPA) contents in the eggs also suggest the activation of the Sprecher pathway, since fish Fads2 have a Δ6 desaturase activity on 24:5n-3, produced by elongation from 22:5n-3, to synthesise DHA after beta-oxidation from 24:6n-3^60^. Such high DHA and n-3 DPA contents in the egg may also be related to an increased mobilization of DHA to ovaries caused by 17β-estradiol, to a lower beta-oxidation of these fatty acids or to morphological changes in the ovaries. In turn, DHA increase in females would lead to an increased production of docosanoids, which play an important role in the induction of oocyte maturation^61^, improving fecundity in terms of eggs produced. Our observation of a direct positive correlation between n-3 LC-PUFA contents in the egg and egg viability and larval survival confirms that these fatty acids are determinant of embryo and larval development^14,43,46,47^.

That the dietary fatty acid profile is reflected in tissue fatty acid profile is well established and our data show that broodstock fed with a diet rich in rapeseed oil led to increased levels of 18C fatty acids and relatively reduced levels of LC-PUFAs in egg lipids. These changes were however mild, where the 18C fatty acids increased by an average 60% in comparison to those from broodstock fed FO and the LC-PUFA were reduced by 30%, while in the RO diet these values were 200% and 20%, respectively in comparison to fish oil based diet. This lower accumulation of 18C fatty acids together with the relatively lower reduction in LC-PUFA in the eggs, in comparison to the diet, suggests an increased LC-PUFA biosynthesis ability in conformity with the trend for an up-regulation of *fads2* expression found in the eggs of broodstock fed RO.

Broodstock diets replacement of FO by VO, namely reduction of dietary LC-PUFA and increase in 18:2n-6 and 18:3n-3, allows the conditioning of gilthead seabream offspring to produce juveniles which can potentially utilize low FM and FO diets better and grow fast^29,31–33^. Total replacement of FO by vegetable oils markedly reduces reproductive performance of gilthead seabream^32,33^, since LC-PUFA are essential for reproduction of this species^30,62^. The partial FO replacement by RO as done here, giving dietary levels for LA + ALA:n-3 LC-PUFA ratio levels of 16:1:16.3 did not negatively affect sperm quality or spawning quality parameters. These results suggest that the LC-PUFA levels in diet RO were able to match the minimum requirements for gilthead seabream broodstock, in agreement with previous studies^30,62^. Thus, the ARA and EPA contents in diet RO were similar and those of DHA even higher than the optimum dietary levels determined for gilthead seabream broodstock^30^. The levels of these LC-PUFA in the present study were also higher than those in broodstock diets used to induce nutritional programming in gilthead seabream, which caused a reduction in all spawning quality parameters^32,33^.

In summary, the results showed that blood *fads2* expression in gilthead seabream broodstock females, which tended to be higher than in males, was positively related to plasma oestrogen levels. Moreover, broodstock with high blood *fads2* expression showed a better reproductive performance, in terms of fecundity and sperm and egg quality, which was correlated with female *fads2* expression. Besides, the present study has demonstrated that it is feasible to reduce ARA, EPA and DHA down to 0.4, 6.6 and 8.4% of total fatty acids, respectively, without affecting spawning quality, in broodstock diets designed to induce nutritional programming effects in the offspring. Further studies are being conducted to test the offspring with low FM and FO diets along life span.

## Methods

### Characterization of broodstock with high or low *fads2* expression (Phase-I)

The broodstock utilized were obtained as part of a series of long-term ongoing selection programmes involving multiple criteria^63,64^. They were reared right from larval stages in our own research facilities of the ECOAQUA Institute. In order to identify broodstock with different ability to synthesize LC-PUFA from LA and ALA, seventy one 2-year old males (1.02 ± 0.38 kg body weight) and one hundred fourteen 4-year old females (2.07 ± 0.39 kg body weight) were individually tagged with PIT tags (EID Iberica SA-TROVAN, Madrid, Spain) and maintained in a 40 m^3^ (5 × 2.35 m) circular tank. The tanks were supplied with seawater (37 g l^−1^ salinity, 17.8–19.0 °C) at a water exchange of 600% daily and maintained under natural photoperiod. Three months before the spawning season, broodfish were fed twice a day for one month with a low FM (5%) and FO (3%) diet (Low FM/FO diet)^15^, high in LA and ALA (Table S4 and S5) and low in LC-PUFA to induce the up-regulation of the fatty acid desaturases 2 gene (*fads2*). After that period, blood samples were collected from all broodfish (n = 185) and centrifuged at 3000×g for 10 min to separate blood cells and plasma. The blood cell samples were stored at − 80 °C until RNA extraction. Seabream broodfish were divided into two categories, namely high (HD) or low (LD), based on their *fads2* mRNA copy numbers per µl in blood cells. So, eighteen fish with the highest *fads2* expression values and eighteen with the lowest ones were selected for the conditioning trial (Phase III). Blood cell *fads2* gene expression level was analysed by droplet digital polymerase chain reaction (ddPCR) as previously described^8,29^.

### Comparison of broodstock quality before feeding the conditioning diets (Phase-II)

The selected males and females from HD and LD broodfish were stocked in twelve (6 HD and 6 LD) 1,000 L fiberglass tanks with a sex ratio of 2 males to 1 female. Broodstock tanks were supplied with 16L min^−1^ filtered seawater (37 ± 0.5‰ salinity) and strong aeration. At the beginning of the spawning season, from 08 January 2018 to 07 February 2018, fish were fed with a commercial diet (Europa Turbot 18, Skretting, Burgos, Spain) (Tables S6 and S5) to ensure that there were no significant differences in the spawning quality among broodfish from the same category (HD and LD). For the evaluation of spawning quality, the spontaneously spawned eggs from each experimental broodstock group were collected four times per week, following this procedure^8,30,33^. Eggs were also collected at the end of the feeding period and kept at − 80 °C until biochemical analysis.

### Broodstock nutritional conditioning (Phase-III)

From 08 February 2018 to 05 April 2018, the twelve broodstock groups from Phase II were fed one of two different broodstock diets (FO or VO diet), under the same conditions described in the above paragraph. The diets were isoproteic and isolipidic, contained either fish oil (FO) or a mixture of 20% fish oil (FO) and 80% rapeseed oil (RO) and were produced by Skretting ARC (Stavanger, Norway) (Tables S6 and S5). Compared to the FO diet, the RO diet had higher levels of 18:2n-6 and 18:3n-3 fatty acids and reduced levels of saturated, monoenoic and n-3 LC-PUFA (20:5n-3; eicosapentaenoic acid, EPA and 22:6n-3; docosahexaenoic acid, DHA) (Table S5). Fish were fed two times a day (9:00 and 14:00 h) at 1% of their estimated total biomass. Seawater temperature during broodstock spawning period was in the range of 18–22 °C (January–April 2018) and fish were kept under natural photoperiod (12 h light). Egg collection for spawning quality and biochemical composition followed the same protocol described in Phase II. Finally, after 30 days of feeding the two different experimental diets, eggs were collected from all broodfish groups (HDFO, HDRO, LDFO, LDRO) and conserved in 1,000 μl of RNA Later (Sigma-Aldrich) overnight at 4 °C, and then samples were kept at − 80 °C until RNA extraction.

### Plasma steroid hormones

At the end of Phase III, all the GSB broodstock were fasted overnight and anesthetized with clove oil (10 ppm clove oil:methanol (50:50) in sea water) to collect blood samples. Blood was taken from the caudal vein using sterile syringes (Terumo Europe NV, Leuven, Belgium) and transferred to 3.0 mL K3-EDTA tubes (L.P. Italiana, Milan, Italy). Whole blood samples were centrifuged at 3,000*g* for 10 min at 4 °C and plasma was separated and stored at − 80 °C for sex steroid hormone analyses. Plasma sex steroids were measured by enzyme immunoassays (EIA) as described for European sea bass for testosterone (T)^65^, 11-ketotestosterone (11-KT)^66^ and 17β-estradiol (E2)^67^ and later validated for seabream^16^. Plasma steroids were extracted with methanol and supernatants were dried and reconstituted in EIA buffer (potassium phosphate 0.1 M, pH 7.4 containing 0.01% sodium azide, 0.4 M NaCl, 0.001 M EDTA and 0.1% BSA). The assays were performed in 96 well plate coated with mouse anti-rabbit IgG monoclonal antibodies (Sigma-Aldrich, R-1008). Steroid standard curves (ranging from 0.0024–5.0 ng/ml for T; 0.0005–1.0 ng/ml for 11-KT and 0.039–80.0 ng/ml for E2; Sigma-Aldrich) or plasma samples were run in duplicate and added to the wells together with the corresponding acetylcholinesterase (AChE) tracer (T-AchE, 11-KT-AChE or E2-AChE; Cayman Chemical, Michigan, USA) and rabbit antiserum (anti-T, anti-11-KT or anti-E2), and incubated at 37 ºC (E2) or 4ºC (T and 11-KT). Next, plates were rinsed, and color development was performed by addition of Ellman reagent. Optical density was read at 405 nm using a microplate reader (Bio-Rad 3550). The sensitivities of the assays (80% of binding) were 0.011 ng/ml for T, 0.0014 ng/ml for 11-KT and 0.31 ng/ml for E2. The inter-assay coefficients of variation at 50% of binding were 10.01% for T, 4.48% for 11-KT and 8.49% for E2. The intra-assay coefficients of variation were 3.78% for T, 3.60% for 11-KT and 1.14% for E2. Sex steroid hormone concentration values are presented as mean ± SD.

### Sperm quality

At the end of Phase III, all the male broodfish were anesthetized as mentioned above and sperm was collected from the blot dried genital pore after a gentle abdominal massage to induce spermiation and taking care to avoid contamination with water, faeces or urine. The collected sperm was stored on ice until transferred to a 4 °C refrigerator. The sperm quality parameters that were evaluated included sperm concentration (number of spermatozoa/ml sperm, 10^9^ ml^−1^), spermatocrit percentage, sperm motility percentage (percentage of spermatozoa showing forward motility) and sperm motility duration (Seconds). Sperm concentration was estimated after a 1,000-fold dilution with sperm inactivation media using a Neubauer haematocytometer under 400× magnification. Sperm motility and motility duration were evaluated on a microscope slide (400× magnification) after mixing 1 µl of sperm with 50 μl of seawater^53,68,69^.

### Egg and larval quality

The collected eggs were placed in 5 l containers provided with aeration, from where 3 randomized 5 ml samples were taken and placed in a Bogorov chamber under the light microscope to calculate the total number of eggs and percentages of fertilized and viable eggs. Egg viability was determined by observing the percentage of morphologically normal eggs after 1-day post fertilization (1 dpf)^8,30^. Then, the viable eggs were individually placed in two replicates in 96-well microtiter plates filled with filtered and sterilized seawater. Eggs were incubated in a controlled temperature incubator at 19–21 °C, to estimate the percentage of hatching (2 dpf) and larval survival rates at 3 days post hatch (dph). From these values, the total numbers of fertilized, viable, hatched and larvae produced per kg female were calculated^8,30^.

### Biochemical analysis

One month after feeding the commercial diet in Phase-II and one month after the experimental conditioning diets in Phase-III, egg samples were collected from all the broodstock groups and stored at − 80 °C for analysis of proximate and fatty acid composition. Crude protein content was determined by measuring the N content (N × 6.25) through automated Kjeldahl analysis^70^ and crude lipid extraction was carried out with chloroform:methanol^71^. Fatty acids from total lipids were prepared by transmethylation^72^ and separated by gas chromatography^73^ and identified by comparison with previously characterized standards and GLC-MS (Polaris QTRACETM Ultra; Thermo Fisher Scientific). Moisture contents were obtained after drying the samples in an oven at 110 °C for 24 h and then for 1 h until constant weight. Ash content was determined after incineration at 600 °C for 16 h.

### Molecular studies

Total RNA from blood cells (300–400 µl) (Phase I) and egg samples (60–70 mg) (Phase III) was extracted using the RNeasy Mini Kit (Qiagen) and homogenized using the Tissue Lyzer-II (Qiagen, Hilden, Germany) with TRI Reagent (Sigma-Aldrich). Samples were centrifuged with chloroform for phase separation (12,000*g*, 15 min, 4 °C). The upper aqueous phase containing RNA was mixed with 75% ethanol and transferred into the RNeasy spin column, where total RNA bound to a membrane and RW1 and RPE buffers (Qiagen) were used to wash away contaminants. Purified RNA was eluted with 50 μL of RNase-free water. The quality and quantity of RNA were analysed using the NanoDrop 1000 Spectrophotometer (Thermo Scientific, Wilmington, DE, USA). Synthesis of cDNA was undertaken using the iScript cDNA Synthesis Kit (Bio-Rad) according to manufacturer's instructions in an iCycler thermal cycler (Bio-Rad, Hercules, CA, USA). The total RNA extraction, cDNA synthesis, primer (*fads2*) designing, and *fads2* gene expression of both blood cells and egg samples were performed as described in our other studies^8,29,32,33^. Primers for *fads2* (Δ6 desaturase) were redesigned in Genetics laboratory of GIA in reference to publications in NCBI (National Center for Biotechnology Information) as follows:

Gene – *fads2* (Δ6 desaturase).

Forward primer sequence: GCA GAG CCA CAG CAG CAG GGA.

Reverse sequence: CGG CCT GCG CCT GAG CAG TT.

### Statistical analysis

Data are reported as mean ± standard deviation. Data were compared statistically using the analysis of variance (ANOVA), at a significance level of 5%. All variables were checked for normality and homogeneity of variance using the Kolmogorov–Smirnoff and the Levene’s tests, respectively. Otherwise, an arcsine transformation was performed to attain normality. When arcsine transformed data were not normally distributed, then Kruskal–Wallis non-parametric test was applied to the non-transformed data. An independent sample student’s *t* test was performed to compare egg biochemical and fatty acid composition during Phase-II to check the broodstock selection (HD or LD) effect. One way and two-way ANOVA were applied to the results of sperm and egg and larval quality parameters (total eggs; fertilized eggs; viable eggs; hatched larvae; 3dph larvae per spawn per kg female and fertilization, egg viability, hatching and larval survival rates), egg biochemical and fatty acid composition of phase-III and egg *fads2* expression to determine the combined effects of broodstock selection (HD or LD) and diet (FO or VO). Linear regression analysis was performed for relationships between specific fatty acids contents in eggs (n-3 LC-PUFA) and egg viability or larval survival (3dph) %. All data were analysed using the program IBM SPSS version 20 for Windows (IBM SPSS Inc., Chicago, IL, USA).

### Ethical statement

The study was conducted according to the European Union Directive (2010/63/EU) on the protection of animals for scientific purposes at GIA, ECOAQUA Institute, University of Las Palmas de Gran Canaria (ULPGC), Canary Islands, Spain. All experimentation performed at the (ULPGC) was approved by the Bioethical Committee of the University of Las Palmas de Gran Canaria (REF: 007/2012 CEBA ULPGC).

## Supplementary information

Supplementary Table

Supplementary Figure

## Data Availability

All data generated or analysed during this study are included in this published article.
