# Supplementary material for: Reproductive performance of gilthead seabream (Sparus aurata) broodstock showing different expression of fatty acyl desaturase 2 and fed two dietary fatty acid profiles
Source: Sci Rep. 2020 Sep 23;10:15547. doi: 10.1038/s41598-020-72166-5 (PMC7512018; doi:10.1038/s41598-020-72166-5)
Supplement: Supplementary file 1 — Supplementary Table [file 41598_2020_72166_MOESM1_ESM.pdf]

Supplementary tables

Reproductive performance of gilthead seabream (*Sparus aurata*) broodstock showing different expression of fatty acyl desaturase 2 and fed two dietary fatty acid profiles

Shajahan Ferosekhan, Hanlin Xu, Serhat Turkmen, Ana Gómez, Juan Manuel Afonso, Ramon Fontanillas, Grethe Rosenlund, Sadasivam Kaushik, and Marisol Izquierdo

Table S1. Quality of egg and larvae obtained from the different gilthead seabream broodstock groups fed with the same commercial diet at the beginning of the spawning season (Phase-II). No significant differences for each spawning quality parameters among broodfish groups (HD *vs* LD) were observed (Independent sample student's *t*-test).

**Egg and larval quality parameters**	**High *fads2* (HD)**	**Low *fads2* (LD)**	***t*-test (*P* value)**
Total eggs/spawn/kg female	51,231 ± 17582	51,203 ± 19038	1.00
Fertilization %	81.35 ± 3.70	79.69 ± 19.02	0.92
Egg viability %	76.77 ± 2.94	72.18 ± 20.56	0.81
Hatching %	81.94 ± 12.74	92.72 ± 1.82	0.32
Larval survival (3dph) %	82.57 ± 4.23	84.44 ± 5.91	0.67

Table S2. Biochemical composition of eggs obtained from the different gilthead seabream broodstock groups fed with the same commercial diet at the beginning of the spawning season (Phase-II). No significant differences for eggs biochemical composition among broodfish groups (HD *vs* LD) were observed (Independent sample student's *t*-test).

**Egg biochemical composition**	**High *fads2* (HD)**	**Low *fads2* (LD)**	***t*-test (*P* value)**
Crude protein (% DM)	67.36 ± 6.03	66.28 ± 2.00	0.77
Crude lipid (% DM)	28.70 ± 2.68	27.95 ± 1.25	0.83
Moisture, %	89.75 ± 0.73	88.87 ± 1.01	0.21

Table S3. Fatty acid profiles (% total fatty acids) of gilthead seabream eggs obtained from the different broodstock groups fed with the same commercial diet at the beginning of the spawning season (Phase-II). Different superscripts in each row would indicate significant differences among broodfish groups (HD *vs* LD) (Independent sample student's *t*-test).

**Fatty acids (%TFA)**	**High *fads2* (HD)**	**Low *fads2* (LD)**	***t*-test (*P* value)**
14:0	1.09 ± 0.21	1.46 ± 0.50	0.22
14:1n-7	0.19 ± 0.15	0.04 ± 0.01	0.15
14:1n-5	0.22 ± 0.17	0.08 ± 0.02	0.21
15:0	0.27 ± 0.11	0.21 ± 0.03	0.46
15:1n-5	0.20 ± 0.13	0.05 ± 0.02	0.11
16:0 ISO	0.23 ± 0.12	0.09 ± 0.03	0.13
16:0	10.45 ± 0.95	12.56 ± 1.60	0.08
16:1n-7	2.79 ± 0.34	3.34 ± 0.60	0.18
16:1n-5	0.25 ± 0.21	0.10 ± 0.02	0.31
16:2n-4	0.27 ± 0.11	0.20 ± 0.03	0.31
17:0	0.26 ± 0.12	0.16 ± 0.02	0.23
16:3n-4	0.31 ± 0.18	0.21 ± 0.00	0.38
16:3n-3	0.28 ± 0.24	0.11 ± 0.02	0.29
16:3n-1	0.30 ± 0.31	0.10 ± 0.03	0.33
16:4n-3	0.36 ± 0.37	0.16 ± 0.03	0.41
18:0	3.17 ± 0.43	3.39 ± 0.28	0.48
18:1n-9	22.41 ± 2.26	25.23 ± 0.82	0.10
18:1n-7	2.66 ± 0.21	2.90 ± 0.06	0.12
18.1n-5	0.27 ± 0.19	0.15 ± 0.02	0.36
18:2n-9	0.26 ± 0.17	0.18 ± 0.06	0.51
18.2n-6 (LA)	11.08 ± 1.02	12.34 ± 0.79	0.14
18:2n-4	0.26 ± 0.11	0.15 ± 0.01	0.14
18:3n-6	0.56 ± 0.36	0.31 ± 0.09	0.27
18:3n-4	0.50 ± 0.25	0.22 ± 0.06	0.12
18:3n-3 (ALA)	2.38 ± 0.20	2.66 ± 0.15	0.10
18:4n-3	0.60 ± 0.10	0.64 ± 0.02	0.49
18:4n-1	0.31 ± 0.15	0.15 ± 0.03	0.12
20:0	0.25 ± 0.12	0.13 ± 0.04	0.16
20:1n-9	0.29 ± 0.09	0.18 ± 0.03	0.11
20:1n-7	1.33 ± 0.12	1.18 ± 0.14	0.20
20:1n-5	0.34 ± 0.09	0.19 ± 0.02	0.06
20:2n-9	0.32 ± 0.21	0.12 ± 0.02	0.16
20:2n-6	0.69 ± 0.15	0.54 ± 0.06	0.15
20:3n-9	0.28 ± 0.24	0.06 ± 0.01	0.17
20:3n-6	0.54 ± 0.32	0.22 ± 0.07	0.17

20:4n-6 (ARA)	0.82 ± 0.05	0.79 ± 0.06	0.57
20:3n-3	0.56 ± 0.28	0.36 ± 0.04	0.27
20:4n-3	0.89 ± 0.06	0.78 ± 0.02	0.06
20:5n-3 (EPA)	5.43 ± 0.66	5.76 ± 0.45	0.49
22:1n-11	0.55 ± 0.22	0.36 ± 0.05	0.19
22:1n-9	0.44 ± 0.24	0.22 ± 0.02	0.16
22:4n-6	0.55 ± 0.43	0.12 ± 0.02	0.14
22:5n-6	0.68 ± 0.54	0.26 ± 0.02	0.22
22:5n-3	3.37 ± 0.35	2.91 ± 0.42	0.17
22:6n-3 (DHA)	20.77 ± 1.28	18.54 ± 2.68	0.20
Σ Saturates	15.48 ± 1.01	17.93 ± 1.85	0.07
Σ Monoenes	31.91 ± 1.34	34.02 ± 1.09	0.08
Σ n-3	34.62 ± 1.39	31.92 ± 3.42	0.20
Σ n-6	14.89 ± 0.93	14.59 ± 0.60	0.64
Σ n-3 LC-PUFA	31.01 ± 1.77	28.35 ± 3.59	0.25
DHA/EPA	3.86 ± 0.37	3.21 ± 0.22	0.04
DHA/ARA	25.47 ± 0.57	23.41 ± 1.94	0.20
n-3/n-6	2.33 ± 0.22	2.20 ± 0.33	0.53

Table S4. Ingredients and proximate composition of the diet for gilthead seabream broodstock used during Phase-I.

Ingredients (%)	Low FM/FO diet
Fish meal[1]	5.00
Blood meal (spray-dried)[2]	7.00
Soya protein concentrate[3]	20.00
Corn gluten meal[4]	22.00
Wheat gluten[4]	5.50
Rapeseed meal[5]	11.30
Wheat[6]	6.89
Fish oil[7]	3.00
Rapeseed oil[5]	5.20
Linseed oil[8]	2.60
Palm oil[4]	5.20
Supplemented ingredients[9]	5.49
Vitamin and mineral premix[10]	0.75
Antioxidant[11]	0.05
Yttrium oxide	0.03
**Proximate composition**	
Crude protein (% dry matter, DM)	45.1
Crude lipid (%DM)	21.7
Moisture (%)	9.0
Ash (%DM)	5.4

[1]South-American, Superprime – Feed Service Bremen, Germany
[2]Daka, Denmark
[3]Svane Shipping, Denmark
[4]Cargill, Netherlands
[5]Emmelev, Denmark
[6]Hedegaard, Denmark
[7]South American fish oil, LDN Fish Oil, Denmark
[8]Ch. Daudruy, France
[9]Contains lysine, methionine, monocalcium phosphate, choline, inositol, phospholipids (Emulthin G35). Vilomix (Denmark), Evonik Industries (Germany), Pöhner (Germany)
[10]Supplied the following vitamins (mg/kg): A 3.8, D 0.05, E 102.4, K3 9.8, B1 2.7, B2 8.3, B6 4.8, B12 0.25, B3 24.8, B5 17.2, folic acid 2.8, H 0.14, C 80; minerals (mg/kg): cobalt 0.94, iodine 0.7, selenium 0.2, iron 32.6. manganese 12, copper 3.2, zinc 67; other (g/kg): taurine 2.45, methionine 0.5, histidine 1.36, cholesterol 1.13. DSM, (Netherlands), Evonik Industries (Germany), Deutsche Lanolin Gesellschaft (Germany)
[11]BAROX BECP, Ethoxyquin, Vilomix (Denmark)

Table S5. Fatty acid profiles of the broodstock diets used in Phases I, II and III (% total fatty acids).

Fatty acid (%TFA)	Phase I Low FM/FO	Phase II Commercial diet	Phase III	
			FO diet	RO diet
14:0	6.60	3.50	5.04	1.87
14:1n-5	0.10	0.10	0.15	0.08
15:0	0.10	0.27	0.46	0.17
16:0 ISO	0.00	0.05	0.09	0.09
16:0	12.30	12.46	18.83	9.42
16:1n-7	2.10	3.76	6.84	2.67
16:1n-5	0.10	0.14	0.26	0.11
16:2n-4	0.20	0.34	0.75	0.29
17:0	0.30	0.28	0.83	0.20
16:3n-4	0.10	0.17	0.23	0.17
16:3n-1	0.00	0.12	0.20	0.11
16:3n-3	0.00	0.08	0.12	0.08
16:4n-3	0.40	0.54	1.09	0.43
18:0	3.20	2.42	3.95	2.47
18:1n-9	32.30	32.48	12.82	31.76
18:1n-7	2.30	3.29	3.37	3.28
18:1n-5	0.00	0.15	0.30	0.16
18:2n-9	0.00	0.02	0.19	0.04
18:2n-6 (LA)	20.30	11.51	4.11	11.14
18:2n-4	0.10	0.08	0.24	0.09
18:3n-6	0.10	0.13	0.32	0.13
18:3n-4	0.00	0.07	0.15	0.14
18:3n-3 (ALA)	11.80	5.10	1.30	4.95
18:4n-3	0.40	1.28	2.19	1.22
18:4n-1	0.00	0.06	0.00	0.11
20:0	0.40	0.49	0.47	0.61
20:1n-9	1.00	3.27	3.77	4.06
20:1n-7	0.10	0.17	0.31	0.18
20:2n-9	0.00	0.02	0.06	0.05
20:2n-6	0.10	0.15	0.20	0.17
20:3n-9	0.00	0.04	0.07	0.09
20:3n-6	0.00	0.04	0.12	0.10
20:4n-6 (ARA)	0.20	0.38	1.04	0.43
20:3n-3	0.00	0.08	0.15	0.12
20:4n-3	0.10	0.25	0.57	0.35
20:5n-3 (EPA)	2.50	5.52	11.96	6.57
22:1n-11	0.10	3.45	3.73	4.98
22:1n-9	0.30	0.48	0.51	0.66
22:4n-6	0.00	0.05	0.17	0.23
22:5n-6	0.10	0.11	0.43	0.27

22:5n-3	0.30	0.49	1.40	0.79
22:6n-3 (DHA)	1.70	6.08	11.11	8.42
Σ Saturates	22.90	19.42	29.58	14.74
Σ Monoenes	38.40	47.75	32.12	48.59
Σ n-3	17.20	19.42	29.89	22.93
Σ n-6	20.80	12.38	6.38	12.47
Σ n-3 LC-PUFA	4.60	12.42	25.19	16.25
DHA/EPA	0.68	1.10	0.93	1.28
DHA/ARA	8.50	15.82	10.72	19.82
n-3/n-6	0.83	1.57	4.68	1.84

Table S6. Ingredients and proximate composition of commercial (Phase-II) and experimental diets (FO and RO) used for broodstock nutritional conditioning (Phase-III).

**Ingredients (%)**	**Commercial diet**	**Exp. Diets**	
		**FO**	**RO**
Fish meal (North-Atlantic 12C)		59.36	59.36
Squid meal		3.00	3.00
Krill meal		7.00	7.00
Wheat		20.57	20.57
Fish oil (South American)		9.30	1.76
Rapeseed oil		0.00	7.54
Vitamin-mineral premix*		0.50	0.50
L-Histidine HCl		0.27	0.27
**Proximate composition**			
Crude protein (%DM)	57.6	53.4	54.6
Crude lipid (%DM)	17.3	18.8	17.3
Ash (%DM)	9.8	11.3	11.6
Moisture (%)	8.9	7.9	7.3

Vitamin-mineral premix*: vitamins (mg/kg): A 3.8, D 0.05, E 102.4, K3 9.8, B1 2.7, B2 8.3, B6 4.8, B12 0.25, B3 24.8, B5 17.2, folic acid 2.8, H 0.14, C 80; minerals (mg/kg): cobalt 0.94, iodine 0.7, selenium 0.2, iron 32.6. manganese 12, copper 3.2, zinc 67; other (g/kg): taurine 2.45, methionine 0.5, histidine 1.36, cholesterol 1.13. DSM, (Netherlands), Evonik (Germany), Deutsche Lanolin Gesellschaft (Germany).
