# Supplementary material for: Reproductive performance of gilthead seabream (Sparus aurata) broodstock showing different expression of fatty acyl desaturase 2 and fed two dietary fatty acid profiles
Source: Sci Rep. 2020 Sep 23;10:15547. doi: 10.1038/s41598-020-72166-5 (PMC7512018; doi:10.1038/s41598-020-72166-5)
Supplement: Supplementary file 2 — Supplementary Figure [file 41598_2020_72166_MOESM2_ESM.pdf]

Supplementary figures

Reproductive performance of gilthead seabream (*Sparus aurata*) broodstock showing different expression of fatty acyl desaturase 2 and fed two dietary fatty acid profiles

Shajahan Ferosekhan, Hanlin Xu, Serhat Turkmen, Ana Gómez, Juan Manuel Afonso, Ramon Fontanillas, Grethe Rosenlund, Sadasivam Kaushik, and Marisol Izquierdo

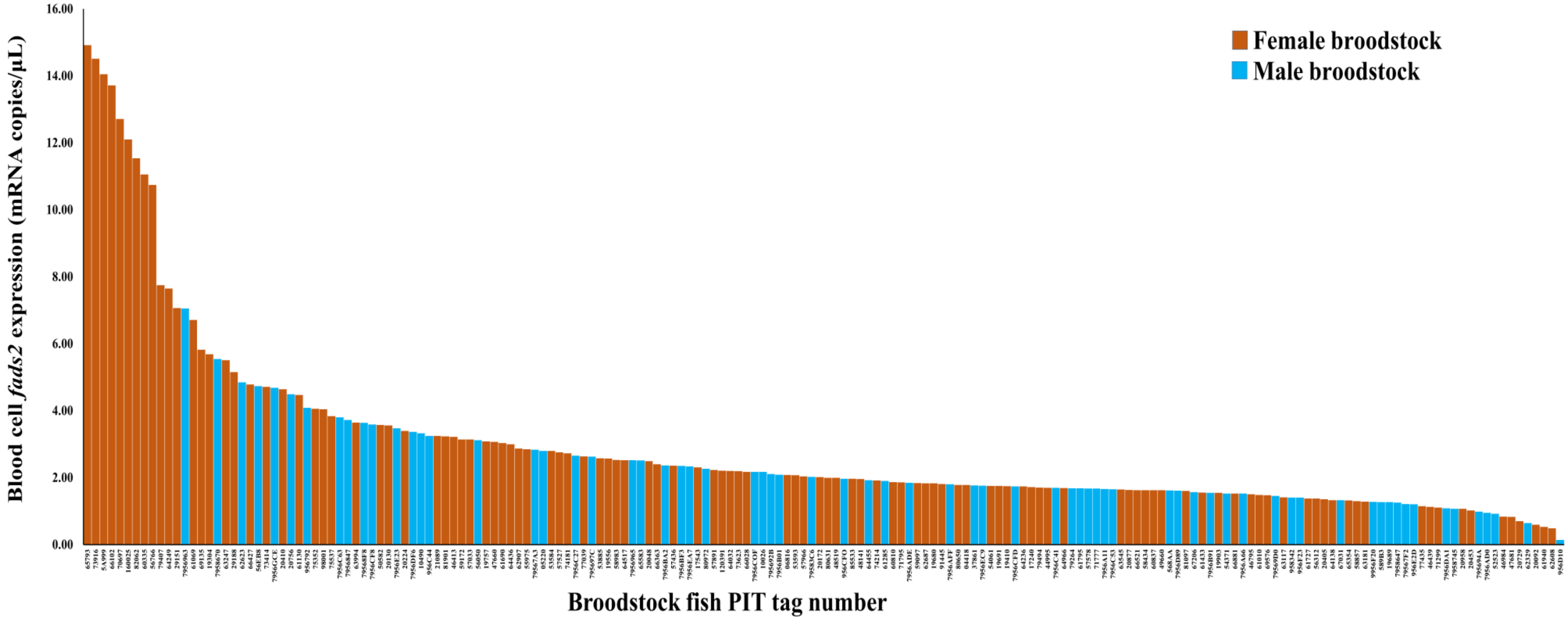

Figure S1. Values of *fads2* (mRNA copies/µL) expression in blood cells of gilthead seabream male and female broodstock after being fed the low FM and low FO diet at the end of Phase I.

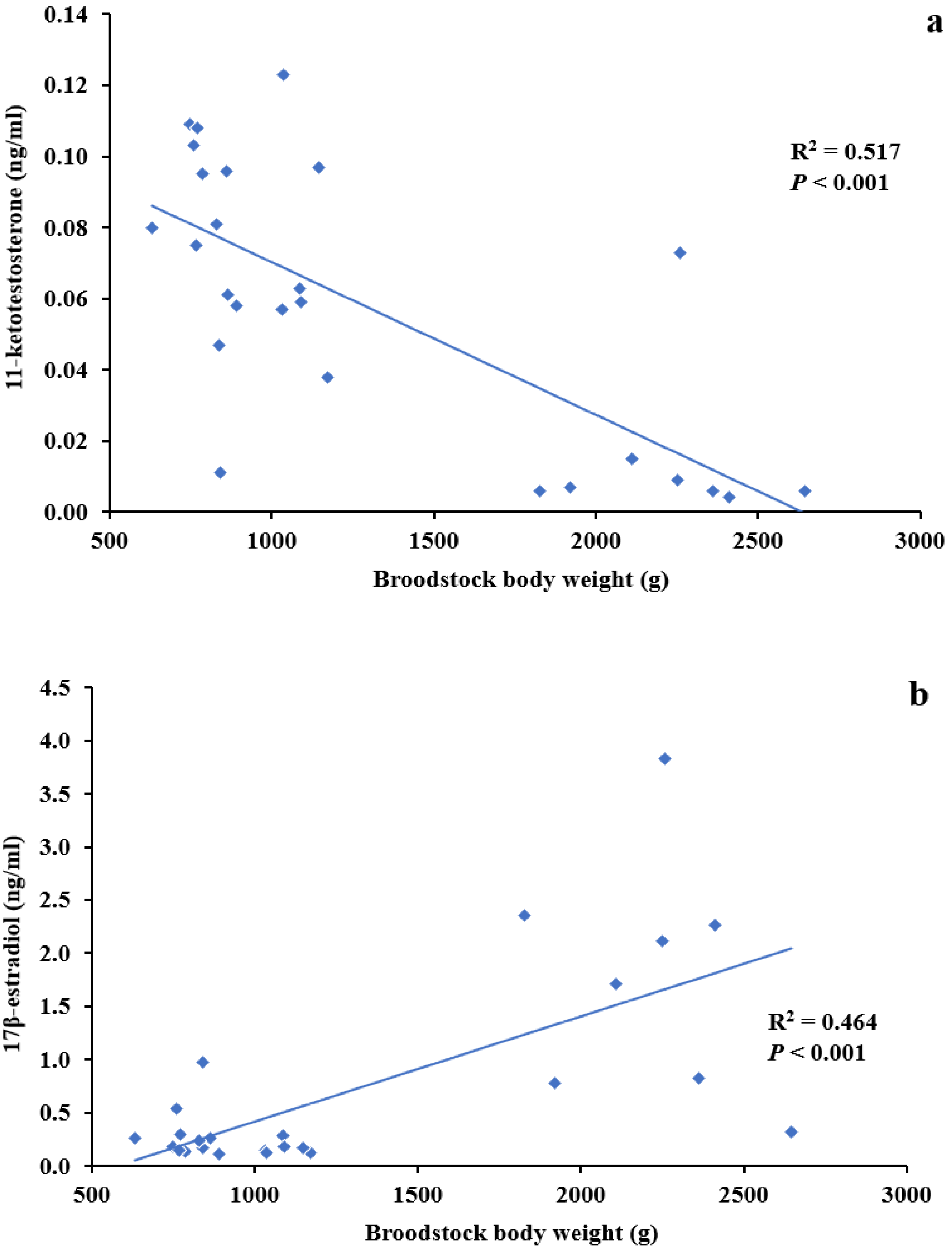

Figure S2. The linear regression relationship between body weight and plasma levels of 11-ketotestosterone (a) or 17β-estradiol (b) in gilthead seabream broodstock at the end of Phase III.
